# A rare coexistence of non-functional adrenocortical carcinoma and multicentric papillary thyroid microcarcinoma: a case report

**DOI:** 10.1186/1752-1947-7-200

**Published:** 2013-07-26

**Authors:** Melia Karakose, Oguz Hasdemir, Erman Cakal, Tuncay Delibasi

**Affiliations:** 1 Department of Endocrinology and Metabolism, Diskapi Yildirim Beyazit Training and Research Hospital, Altindag, Ankara, Turkey; 2 Department of General Surgery, Diskapi Yildirim Beyazit Training and Research Hospital, Altindag, Ankara, Turkey

**Keywords:** Adrenocortical carcinoma, Papillary thyroid carcinoma, Hormone

## Abstract

**Introduction:**

In this report, we describe a rare case of papillary thyroid carcinoma with adrenocortical carcinoma without excess hormone production.

**Case presentation:**

A 40-year-old Turkish man was admitted to our institution with a large left adrenal mass that was identified during the work-up for shortness of breath. The patient did not have specific signs and symptoms of hormone excess. The mass was removed surgically. The pathological findings were consistent with adrenocortical carcinoma. The patient was also found to have a multicentric papillary thyroid microcarcinoma.

**Conclusion:**

Most adrenocortical carcinomas and papillary thyroid carcinomas are sporadic; however, the occurrence of two different endocrine neoplasms during the same period of time is a rare situation, but it is possible, as in our patient. When an endocrine tumor is diagnosed, endocrinologists must be consider the possibility of the existence of another endocrine tumor.

## Introduction

Adrenocortical carcinomas (ACCs) are an extremely rare type of cancer with an incidence of less than 2 per 1 million worldwide [1,2]. Approximately 60% of patients present with symptoms of excess hormone secretion or manifest themselves as symptoms and signs due to mass effect. Thyroid cancer is the most common malignant endocrine tumor. Its annual incidence varies between 0.5 to 10 per 100,000. Papillary thyroid carcinoma (PTC) is the most common form of differentiated thyroid carcinoma and is more common in women than in men. It is often diagnosed during investigation of a thyroid nodule in clinics.

There are few case reports in the literature that describe comorbid ACC and PTC. The available reports attribute the concomitant appearance of the tumors to coincidence and do not discount a potential genetic or hereditary link [3-5]. Herein we report a rare coexistence of PTC and ACC without clinical hormone excess.

## Case presentation

**Figure 1:**
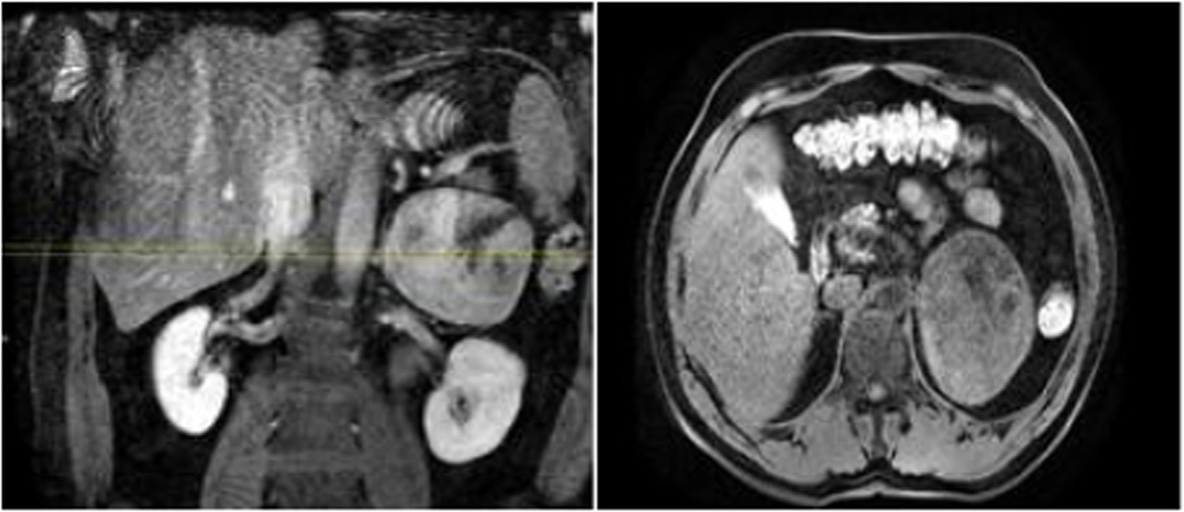
Dynamic magnetic resonance imaging scans (left adrenal mass).

A 40-year-old Turkish man who presented to our institution with shortness of breath was evaluated by computed tomography. A mass in his left adrenal gland was diagnosed incidentally. A magnetic resonance imaging (MRI) scan showed a large mass measuring 8×9.5×10.4cm in diameter in the left adrenal gland area (Figure 1). His serum cortisol, adrenocorticotropic hormone, dehydroepiandrosterone sulfate, total testosterone, urinary metanephrine, serum electrolytes (Na^+^, K^+^,Cl^–^), fasting blood glucose, and fasting insulin levels were normal. Plasma cortisol was suppressed after administration of 1mg dexamethasone. The patient was diagnosed with a non-functional adrenal mass, and a left adrenalectomy was performed. The resected tumor was 11×9×6cm in size and weighed 339g. The tumor was of high nuclear grade and had diffuse architecture, focal necrotic areas, more than 5 mitoses per 50 high-power fields, and an infiltrated capsule. Sinusoidal vascular infiltration was observed, but clear cells were less than 25%. Immunohistochemical studies showed that melan-A and inhibin were positive, and thyroid transcription factor-1 (TTF-1) and thyroglobulin were negative. The pathological findings were consistent with ACC. Treatment with adjuvant radiotherapy with concurrent mitotane was planned for the patient.

Evaluations of thyroid function tests were normal before treatment and anti-thyroglobulin antibody was positive, but anti-thyroperoxidase antibody was negative. Ultrasonographic evaluation of the thyroid revealed the following: There was a nodule 3×4×4mm in size in the lower part of the left lobe and foci of microcalcification, and the edges were irregular and of mixed echogenicity.

**Figure 2:**
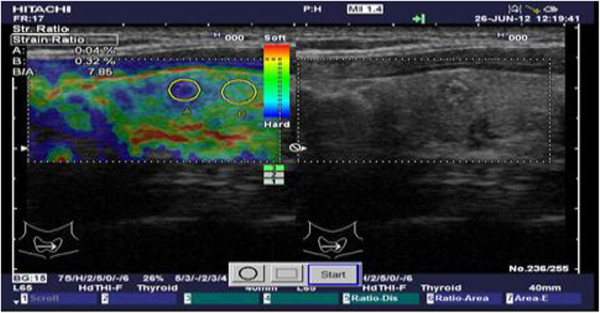
Elastosonographic and ultrasound images of the thyroid nodule.

The elastosonography score of the nodule was 5, and the strain index was calculated as 7.85 (Figure 2). The patient had neither a history of radiation exposure nor a family history positive for thyroid carcinoma. A fine-needle aspiration biopsy of the nodule was suspicious for malignancy. The patient underwent total thyroidectomy and central neck dissection. Tumors were detected in two foci with diameters of 0.9cm and 0.4cm in the left lobe. There was neither lymphovascular nor capsular invasion or lymph node metastasis. Immunohistochemical studies showed that thyroglobulin was positive. The pathologic diagnosis was multicentric papillary thyroid microcarcinoma.

## Discussion

ACCs are a rare type of malignancy (incidence of 1 to 2 cases per 1 million population) with a heterogeneous presentation and generally have a poor prognosis [1,6]. Women are affected more often than men [7,8]. The diagnosis of malignancy of adrenocortical tumors depend on careful investigations of clinical, biological, and imaging features before surgery and pathological examination. The assessment of the Weiss score is important for the diagnosis of malignancy [9,10]. Adrenocortical tumors might be seen as a component of several hereditary tumor syndromes such as Li-Fraumeni syndrome, Beckwith-Wiedemann syndrome, multiple endocrine neoplasia 1, Carney complex, and congenital adrenal hyperplasia. A lot of specific genes might have a role in the pathogenesis of sporadic ACC, and some of them might be seen in the pathogenesis of the aforementioned syndromes [11,12].

Patients present with evidence of adrenal steroid hormone excess in approximately 60% of cases. Rapidly progressing Cushing’s syndrome with or without virilization is the most frequent presentation. Hormonal inactive ACC usually presents with abdominal discomfort (nausea, vomiting, and abdominal fullness) or back pain caused by a mass effect of the large tumor.

PTC comprises about 85% of cases with differentiated thyroid carcinoma [13]. PTC usually presents with a thyroid nodule or cervical lymphadenopathy. Nodules more than 1cm in diameter have a greater potential to be malignant. On the other hand, patients with nodules less than 1cm in diameter, suspicious ultrasound findings, lymphadenopathy, a history of neck and head irradiation, or a history of thyroid cancer in first-degree relatives must be evaluated for cancer [14].

The ultrasonographic features suggesting the presence of malignancy in a thyroid nodule have been described clearly. These include microcalcifications, marked hypoechogenicity, absent “halo” sign, extraglandular extension, an irregular or microlobulated margin, and a heterogeneous echo structure [15,16]. Individual sonographic features have limitations for prediction of thyroid cancer. Elastosonography is a newly developed, promising sonographic method for evaluating suspicious nodules. It gives information about the stiffness of a nodule by measuring the amount of distortion that occurs when the nodule is subjected to external pressure [17]. Tissue stiffness is scored from 1 to 5 based on subjective analysis of the elastographic image. Rago *et al.* reported sensitivity of 97% and specificity of 100% for a score of 4 or 5 as being predictive of malignancy [18].

PTC generally metastasizes to the lymph nodes. Distant organ metastasis is rare, but lung, bone, brain, liver, and adrenal gland metastases have been reported [19,20]. ACC may metastasize to the thyroid gland [21]. Immunohistochemical staining is used to differentiate the primary and metastatic tumors. Melan-A, inhibin, and some other markers have been found to be positive in patients with ACC [22-24], and thyroglobulin and TTF-1 have been found to be positive in patients with thyroid neoplasms in immunohistochemical studies [25-28]. In our case, melan-A and inhibin were positive, but thyroglobulin and TTF-1 were negative, in the patient’s adrenal gland tumor. The thyroid lesion was thyroglobulin-positive. Therefore, these lesions were evaluated as primary tumor neoplasms of those organs.

In the literature, there are few case reports of patients with with comorbid ACC and PTC. Fukushima *et al.* reported a case of a middle-aged woman with virilizing adrenocortical adenoma complicated with Cushing’s syndrome, PTC, and hypergastrinemia [3]. They did not study genetic analyses in their patient. Wanta *et al.* reported the case of a patient with synchronous presentation of an aldosterone-secreting metastatic ACC and PTC [4]. That patient was diagnosed as having Li-Fraumeni syndrome and multiple endocrine neoplasia type I, but both *p53* and menin gene mutations were absent.

## Conclusion

Herein we present a case of a patient with PTC and ACC without clinical hormone excess. Most ACCs and PTCs are sporadic; however, the possibility of a hereditary cancer syndrome could not be ruled out because of the co-occurrence of two different endocrine neoplasms within the same period of time. Therefore, when an endocrine tumor is diagnosed in a patient, endocrinologists must consider the possibility of the existence of another endocrine tumor.

## Consent

Written informed consent was obtained from the patient for publication of this case report and any accompanying images. A copy of the written consent is available for review by the Editor-in-Chief of this journal.

## Competing interest

The authors declare that they have no competing interests.

## Authors’ contributions

MK analyzed and interpreted the patient’s data. The surgery was performed by OH. EC and TD contributed to the writing of the manuscript’s Discussion section. All authors read and approved the final manuscript.
